# Motorizing the buckled blister for rotary actuation

**DOI:** 10.1002/EXP.20230055

**Published:** 2024-03-14

**Authors:** Pengfei Yang, Ruixing Huang, Fei Dang, Baoxiang Shan, Dewen Wang, Hong Liu, Yi Li, Xiangbiao Liao

**Affiliations:** ^1^ School of Mechanical Engineering and Automation Fuzhou University Fuzhou China; ^2^ State Key Laboratory of Explosion Science and Technology and Advanced Research Institute of Multidisciplinary Science Beijing Institute of Technology Beijing China; ^3^ College of Physics and Electronic Information Engineering Minjiang University Fuzhou China; ^4^ Yangtze River Delta Graduate School of Beijing Institute of Technology Jiaxing China; ^5^ Swave Biotechnology (Suzhou) Co. Ltd Suzhou China; ^6^ Department of Materials Science and Engineering University of Connecticut Storrs Connecticut USA

**Keywords:** buckling, rotary actuation, rotary mechanism, snap‐through bistability

## Abstract

Snap‐through bistability was widely exploited for rapid hopping in micro‐electro‐mechanical systems and soft robots. However, considerable energy input was required to trigger the transition between discrete buckling states blocked by potential wells. Here a dynamic buckling mechanism of a buckled blister constrained inside an outer ring is explored for eliciting rotary actuation via a localized change of curvature in the blister. Due to rotational invariance of the buckled blister, lower energy supply is required to initiate the snap‐through of buckling compared to conventional bistable mechanism. The controllability in rotational speed and output torque of the bimetallic blister‐based rotator inside a rigid stator is exhibited, and the locomotion is demonstrated with two elastic rings via localized pneumatic actuators. With broad choices of stimulus and material for rings, the findings illustrate the promising potential of two nested rings to create active motions for diverse applications including gearless motors, peristaltic pumps, and locomotive robots.

## Graphical abstract

We explore the rotation of a buckled blister constrained inside an outer ring. The rotation of buckled blister iss actuated via a localized change of curvature, and lower energy‐supply is&amp;#x000A0;required to initiate the snap‐through of buckling compared to conventional bistable mechanism. We experimentally demonstrate the controllability in rotational speed and output torque for a bimetallic blister‐based rotator inside a rigid stator.

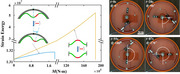

## 1 INTRODUCTION

Buckling, generally avoided in classical structural designs,^[^ ^1^ ^]^ has been yet designed to reconfigure materials and generate complex structures.^[^ ^2^ ^]^ The dynamics of buckling subjected to external stimuli has been attracting more attentions in actuators^[^ ^3^ ^]^ and soft robotics,^[^ ^4^ ^]^ while its quasi‐static behavior was extensively studied. The snapping instability of buckles underpins a promising actuation strategy of triggering rapid deformation via a slow input stimulus. Once external works overcome the potential well, a dynamic transition occurs between discrete buckling morphologies. This strategy has been widely exploited to make micro‐valves/switches,^[^ ^5^ ^]^ deformable metamaterials,^[^ ^6^ ^]^ and inflatable soft jumpers.^[^ ^7^ ^]^ However, the transient state was energetically unfavorable, making the abrupt snapping hardly control, and significant input energy was required to induce the transition between discrete buckling states.^[^ ^7a^ ^]^ Recently, continuous snap‐throughs under steady excitation were investigated in the constraint‐based feedback loops^[^ ^8^ ^]^ and the biological system of cell cortex,^[^ ^9^ ^]^ generating travelling waves.^[^ ^10^ ^]^ The mechanism underpinned controllable actuations and cyclic motions, which has been employed in self‐oscillating systems and light‐driven locomotion devices.^[^ ^11^ ^]^ Nevertheless, due to the main component of soft polymers, small output forces or torques in such systems, much smaller than that of traditional electrical motors, challenged practical applications for actuations.

Elemental motions including bending and rotation are significant for designing machines and robotics. Extensive advances in pneumatic‐based bending actuators presented diverse capabilities of grasping,^[^ ^12^ ^]^ crawling,^[^ ^13^ ^]^ walking,^[^ ^4^ ^]^ and rolling^[^ ^14^ ^]^ for soft robots. Pneumatic actuators realized complete rotation through locomotors,^[^ ^15^ ^]^ but hardly delivered torque through simple rotation. Conventional robots typically adopt mechanical motors having a complex rotor‐stator system. It was unavoidable to incorporate additional multiple gears to realize operations at required rotational speeds. The pioneering system of pneumatically driven inflatable stator paring with a rotor has been fabricated to serve as a rotation‐based wheeled robot, but it required sophisticated structures and precise control of pneumatic pressure.^[^ ^15^ ^]^ Thus, a gearless rotary actuator with simple structures and compatible materials is desirable for practical applications.

**FIGURE 1:**
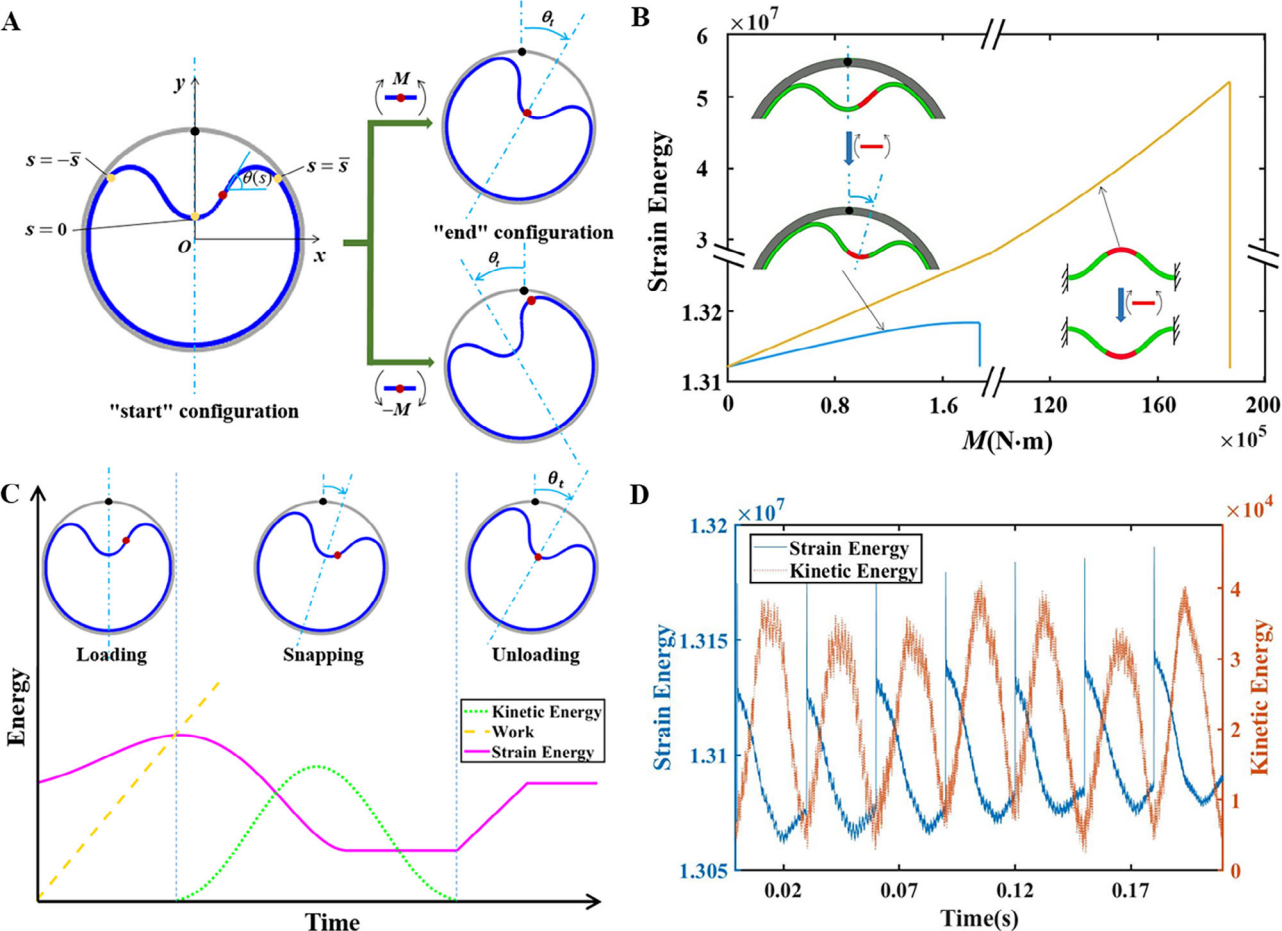
Mechanism schematics of motorizing a buckled blister. (A) The “start” and “end” configurations (blue) represent positions ($\theta_{t}$) of the buckled blister before and after applying a local bending moment (M). A reversed moment resulted in an opposite rotation of the blister. The zero‐curvature point (ZCP) of blister is marked by the dark red point in the “start” configuration. (B) The changes in strain energy with increasing local bending moment, for the buckled blister constrained by a rigid outer ring (blue line) and the bistable beam with built‐in boundaries (yellow line). The material and configuration of such two buckles share the same property and geometry. (C) The transformations of kinetic and strain energies in the inner ring with loading and unloading an increasing torque. The rotation of buckled blister accompanied with the occurrence of snapping. (D) The cyclic transformations of kinetic and strain energies in the inner ring with periodically applying the bending moment in FEM simulations. The unit of energy is joule.

Here, we develop a simple structure of constraint buckled blister for rotational actuation. The buckled blister is formed via confining a closed beam (length $L_{1}$) inside another ring with a smaller circumference $L_{2}$ shown in Figure 1A. We further uncover the dynamic mechanism for motorizing the buckled blister to travel in the circumferential direction via a local bending torque. The localized torque $M(s_{0})$ can be induced by varieties of external stimuli (e.g. thermal, pneumatic, and piezoelectric). The blister circumferentially travels in either the clockwise or the anti‐clockwise direction via controlling the direction of torque, where $s_{0}$ denotes the position of applied stimulus, marked by a red point in the blister. Compared to the conventional bistable beams constrained by built‐in boundaries, lower level of energy supply and smaller threshold of external torque are required to trigger the rotational snap‐through of a buckled blister inside a rigid outer ring. The rotationally traveling blister keeps a mechanically frustrated dynamic steady state,^[^ ^16^ ^]^ showing a global zero‐elastic‐energy mode due to the rotational symmetry of blister around the center of outer ring.

## 2 RESULTS

### 2.1 Driving mechanism of buckled blisters

The inner ring conforms to the wall of outer ring in major regions, but energetically buckles to a symmetric blister with a length of $2\bar{s}$. The ratio of mismatched length $\alpha =L_{1}/L_{2}$ dominates the static configuration of buckled blister, and the relative stiffness of beam to that of outer ring $\beta =K_{1}/K_{2}$ also takes effect in two nest rings.^[^ ^17^ ^]^ Compared to the conventional bistable beams constrained by built‐in boundaries, lower level of energy supply and smaller threshold of external torque are required to trigger the rotational snap‐through of buckled blister inside a rigid outer ring (Figure 1B). The energy supply and critical external torque for conventional bistable beam with built‐in boundaries are 4.08×10^7^ J and 1.875×10^7^ N·m, while those values for the proposed buckled blister are 8.3×10^4^ J and 1.811×10^5^ N·m, respectively. The movable boundary results in the rotational invariance in configuration of buckled blister, and the strain energy is conserved while the blister rotating around the ring's center. In contrast, the buckling configurations are discrete in the bistable system with built‐in boundary, and the transition from one stable position to the other is locked by large potential wells.

With increasing the torque, the local deformation and strain energy accumulate, finally breaking the symmetry of buckled blister and initiating the snap‐through of buckling once $M(s_{0})$ exceeds a certain threshold (Figure 1C). The snapping of blister results in a sudden decrease in strain energy and an emergence of kinetic energy. The blister travels into a new position marked as the configuration $\theta_{t}$, while conserving the initial configuration. These energy changes were verified by finite element simulations (FEMs) via applying a thermally induced local torque at the zero‐curvature point (ZCP) of blister (Figure S1). FEMs further demonstrate that when a periodic torque load applying at the ZCP of each new blister configuration $\theta_{t}$, the buckled blister can continuously travel, generating a stable rotation (Movie S1). A periodic change of strain energy is displayed during the blister rotating, and the kinetic energy is periodically undulated with the same frequency but a phase delay of $90^{\circ }$ (Figure 1D). The blister rotation was maintained due to the steady input of thermal stimulus.

**FIGURE 2:**
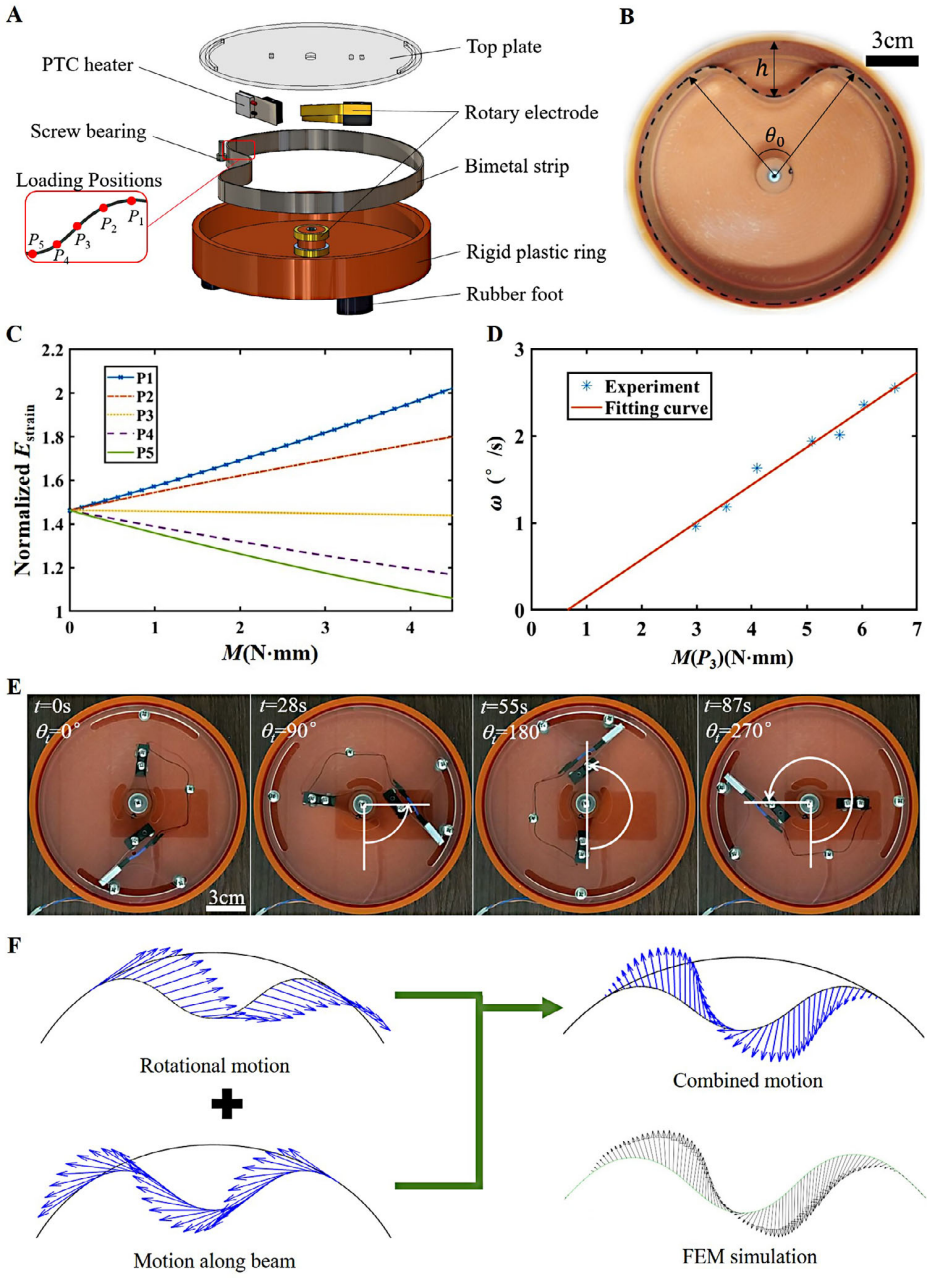
Driving and rotation of the buckled blister. (A) Layer‐by‐layer illustration of the rotating device with a closed bimetal strip (length $L_{1}=50.79\;\mathrm{cm}$, thickness t=0.2mm, width b=1.5cm) confined in a rigid plastic ring (radius 8.0 cm). The PTC heater is electrically connected with a power supply via the rotary electrode. Upon heating at the regions of buckled blister ($P_{1}-P_{5}$), the blister rotates around the ring's center. The screw bearing transferred the rotating motion to the top plate. (B) Geometric comparison between the calculated configuration and the experimental image of buckled blister at equilibrium. $\theta_{0}$ and h denote the characteristic sizes of the blister, and the position of $P_{1}-P_{5}$ are marked. (C) Plots of strain energies (normalized by $K_{1}/b$) of the inner ring with respect to the amplitude of bending moment at different loading positions. (D) Experimentally measured rotation speeds of the blister with the amplitude of applied torque at the position $P_{3}$, which displayed a linear scaling (red line). (E) Images illustrating a stable rotation with a heating temperature of $138^{\circ }C$ at $P_{3}$. (F) Distributions of analytically (Eq. (S1)) and numerically calculated speeds in the travelling blister. The blister speed could be divided into two components of the rotational motion around the ring's center and the linear motion along the beam of inner ring.

To demonstrate the rotation of buckled blister, we fabricate a rotator device consisting of a closed bimetal strip confined in a rigid plastic ring. Figure 2A provides a layer‐by‐layer illustration of the device. The positive temperature coefficient (PTC) heater as the external thermal stimulus was used to induce local torque in the strip made of alloy bilayer, and the loading position $s_{0}$ could be adjusted between $P_{1}$ and $P_{5}$. The rotation can be synchronously transferred to the top plate through a screw bearing. The radius of outer ring was r=8.0 cm and the ratio of mismatched length α=1.011. Due to great compression in the strip, a buckled blister is inwardly formed instead of a completely adhered configuration. The blister exhibits a hump height h=3 cm and an angle of $\theta_{0}=84.36^{\circ }$ for the non‐adhered region (Figure 2B).

We develop an analytical model to evaluate the static configuration of such inward blister. Considering the linear elasticity of strip beam and local external torque, the governing equation for the blister can be:^[^ ^18^ ^]^

(1) $$K_{1}{\left(\theta^{\prime }\left(s\right)+\frac{M\left(s\right)}{K_{1}}\right)}^{\prime }-F_{x}\sin\theta \left(s\right)+F_{y}\cos\theta \left(s\right)=0$$

where $K_{1}=E_{1}\;I_{1}$ is the bending stiffness, and $E_{1}$ and $I_{1}$ represent the elastic modulus of the elastic strip and the moment of inertia, respectively. θ(s) and $s\in [-\bar{s},\;\bar{s}]$ denote the deflection angle relative to the horizontal axis and the arc of blister. $F_{x}$ and $F_{y}$ respectively represent the internal forces in the x and y direction. The applied torque $M\left(s\right)=M_{0}$ with $s\in [s_{0}-\Delta s/2,\;s_{0}+\Delta s/2]$, otherwise M(s)=0. Δs denotes the width of loading region with the center $s_{0}$. At the end point $s=\bar{s}$ of the blister, $\theta^{\prime }\;\left(\bar{s}\right)=1/r$. Through solving this equation with no external torque, the calculated geometric configuration (dash line) matched well with the experimental result (Figure 2B).

We further evaluate the stability of buckled blister subjected to an external stimulus of torque M(s). The simulation result confirms that the snapping of buckled blister results in changing the loading position in the “start” configuration to the maximum curvature point in the “end” configuration (Figure S2). This process minimizes the strain energy of blister strip. The strain energy $E_{\mathrm{strain}}\left(M\left(s\right)\right)=\frac{1}{2}K\int_{-\bar{s}}^{\bar{s}}{\left(\theta^{\prime }\left(s\right)\right)}^{2}ds$ of the “start” static configuration is calculated with fixing the direction of torque. Figure 2C shows the normalized energies $E_{\mathrm{strain}}$ as a function of the torque M at various points $P_{1}-P_{5}$. With increasing the torque amplitude, the strain energies for $P_{1}-P_{3}$ increase in an approximate linear trend, which underpins that there is an energy barrier to be overcome before the blister snapping. On the other hand, the strain energies for $P_{4}-P_{5}$ linearly decrease with increasing the torque. We find that the strain energy decrease in turn when the loading position changes from $P_{1}$ to $P_{5}$ with a certain torque. It is energetically favorable for the loading position ($P_{1}-P_{4}$) in “start” configuration moving to the maximum curvature point ($P_{5}$) in the “end” configuration. The blister configuration locally deforms but maintains static when loading positions at $P_{5}$ (Figure S3).

Experimentally, a critical temperature of $52^{\circ }C$ is found for motorizing the bimetal blister when the PTC heater is placed at $P_{2}$. According to the mechanical property of bimetal, this temperature corresponds to a torque of 1.5N·mm. This local torque is large enough to overcome the energy barrier and allows the blister snapping to happen. Furthermore, the critical temperature and torque for $P_{3}$ close to the ZCP are $47^{\circ }C$ and 1.22N·mm, respectively, lower than those values for $P_{2}$. But no snapping is observed for $P_{1}$ even at the upper limit of heater temperature (60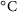, 1.95N·mm). It is demonstrated that the loading position affects the critical torque, and a smaller torque is needed to start the blister rotation when the loading position is close to the ZCP. Controlling and predicting the kinetics of such a blister‐based rotator are of great significance for practical applications. The rotation speed is able to be tuned by the amplitude of local torque M, realized by tuning the heating temperature (Figure 2D). Once the applied torque exceeding the threshold value of 1.22N·mm, the rotation speed exhibits a linear relation with M. The relation is fitted by the expression ω=0.43M. We experimentally demonstrate the rotary motion with a continuous stimulus of $138^{\circ }C$ through fixing the PTC heater at $P_{2}$, and achieve an average angular speed of $3.1^{\circ }$ per second (Figure 2E, Movie S2) in the anti‐clockwise direction.

Furthermore, a numerical model based on finite element analysis is developed to study the dynamic mechanism of the buckled blister. We mimick the continuous rotation of the blister via applying thermal stimulus. The static configuration of blister in geometry matches well with the experimental result (Figure S4). The stress distributions of the buckled and transient states are captured and clearly visualized (Figure S5). It is verified that the stress is not fully relaxed in the transient state, and the breaking of stress symmetry drives the blister circumferentially travelling. The simulations also prove the significance of keeping an uninterrupted stimulus at the blister, which maintains the continuous occurrence of snap‐through and blister rotating (Movie S3). Otherwise, the rotation is suspended by the disappearance of external stimulus due to the energy barrier yet to be overcome. The velocity distribution of blister during rotation is studied. The travelling of buckled blister is composed of a pure rotation (ω) and a linear motion ($v_{m}$) along the beam, but the relation between ω and $v_{m}$ is constrained by the static condition at the detachment point $s=\bar{s}$ due to the assumption of inextensibility and no slipping in two rings. Here, we map the distributions of rotational velocity, linear velocity and their combined velocity along the blister shown in Figure 2F. The result of absolute velocity is also verified by FEM simulations.

### 2.2 Blister‐based rotary motor

**FIGURE 3:**
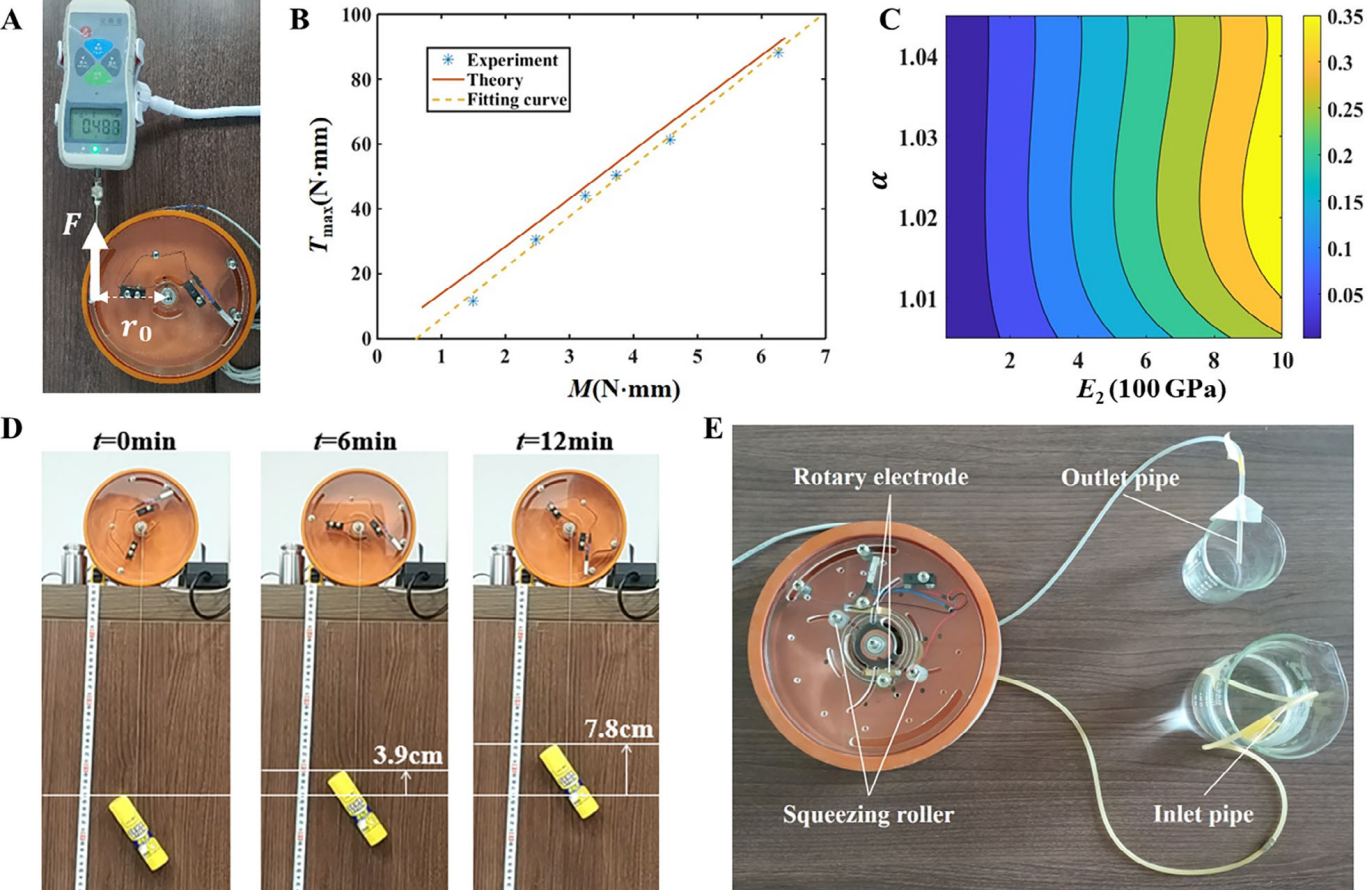
Rotary motor using the buckled blister. (A) Image illustrating the measurement of stalling torque of rotary motor with a heating temperature of $138^{\circ }C$ at $P_{2}$. The rotary motor shared the same device shown in Figure 2A. The rotation of device is balanced by a tensile force of F=1.26N, and the force gauge is placed at the distance of $r_{0}=7\;\mathrm{cm}$ from the outer ring's center. (B) The theoretically calculated and experimentally measured stalling torques as a function of the applied local bending moment at $P_{3}$. Both of theoretical curve (solid line) and fitting curve (dash line) show a linear relation. (C) The theoretically calculated diagram of stalling torque with respect to the modulus $E_{2}$ of inner ring and the ratio of mismatched length α. The amplitude and position of applied torque are fixed. (D) The rotary motor lifting a payload (weight 59 g), where the weight position with increasing time is marked. The device is driven by a thermal stimulus of $138^{\circ }C$ at $P_{3}$. (E) Application demonstration of peristaltic pump using the blister‐based rotary motor. As the blister rotating, water inside the rubber tube is squeezed, flowing from the inlet to the outlet.

Both driving mechanisms and controllable rotation in the buckled blister set forth new applications in mechanical motor, autonomous cargo‐transport, locomotive robotic and peristaltic devices. Here, we demonstrate the capability of as‐fabricated blister‐based rotator to output torque as a mechanical motor. The stalling torque $T_{max}$ of this motor is measured by placing a tensile force gauge F at the screw on the top plate (radius $r_{0}$) until the rotation is suspended while the local thermal stimulus is kept (Figure 3A). A stalling torque of $T_{max}=F\cdot r_{0}=$ 88.2 N·mm is achieved with a heating temperature of $138^{\circ }C$. We further experimentally and theoretically study the effect of applied torque (M) in the stalling torque. As shown in Figure 3B, below the external torque threshold, no torque is exerted, and the onset is discontinuous due to the energy barrier for blister snap‐through. The stalling torque linearly increases with the stimulus amplitude, fitted by the expression $T_{max}=15.75\;M$. It is found that the local torque is amplified by more than 15 times through this blister‐based motor. The high stalling torque stand out when compared to other reported soft rotators driven thermally or pneumatically.^[^ ^15^ ^]^

Additionally, $T_{max}=F_{b}\cdot r_{b}$, where $F_{b}$ is the interaction force between the screw bearing and the bimetal blister, and $r_{b}$ is the distance from the ring's center to the bearing. Through incorporating the external force $F_{b}$ into the balancing Equation (1), the calculated values of $T_{max}$ are illustrated in the solid line (Figure 3B). The theoretical prediction exhibits a linear trend, but is slightly different from the experimental result. The overestimation by the analytical model is due to losing sight of damping and friction in the blister‐based motor. We further theoretically predict the tunability of stalling torque by varying geometrics and properties (Supporting Information). With fixing the applied local torque, we map the stalling torque with respect to the ratio of mismatched length α and the modulus of confined strip $E_{2}$ in Figure 3C. The stalling torque is highly dependent on the modulus, while the mismatched length has little effect in the stalling torque. This self‐contained and simple rotary motor exhibit a controllable function of torque amplifier.

We demonstrate practical applications of such blister‐based rotary motor. The capability of this motor to lift a macroscopic load of 59 g is exhibited in Figure 3D. At a thermal stimulus of $138^{\circ }C$, the load is stably lifted at a constant speed of 0.65 $\mathrm{cm}\;\min^{-1}$ (movie S4). To utilize the stable rotation with a low speed of 0.24 rpm, a peristaltic device for pumping water is fabricated with two squeezing rollers fixed on the top plate (Figure S6, Movie S5). The blister‐driving rotation pushes forward the rolling of such roller, by which an elastic pipe filled with liquid was synchronously pressed. Thus, water is continuously pumped at a flow rate of 0.05 $\mathrm{mL}\;\min^{-1}$.

### 2.3 Rolling‐based locomotor of two elastic rings

**FIGURE 4:**
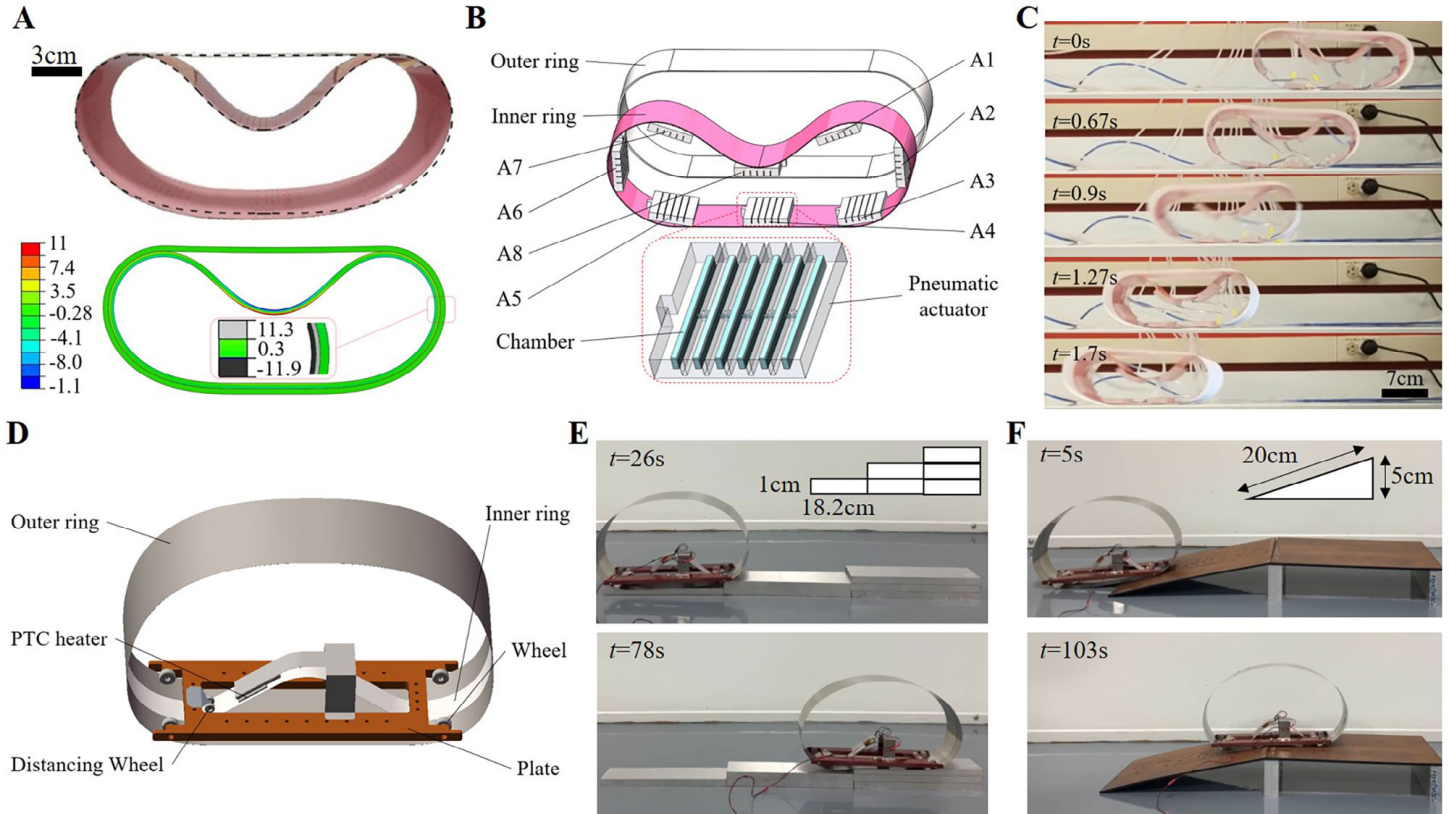
Rolling‐based locomotor of two elastic rings. (A) Geometric comparison between the calculated configuration and the experimental image of two elastic loops (inner loop: PVC, outer loop: printing paper) vertically placed on a flat table (upper). Distribution of stress in the two loops (unit: GPa); Inset: stress distribution in the magnified region of blister apex. In the FEM simulation, gravity is applied to the whole model. (B) Layer‐by‐layer illustration of the wheeled device with two nested loops locally driven by pneumatic actuators, which periodically adhere along the inner loop. The structures of chambers inside actuators are well‐designed to provide bending moments to the blister of inner loop. (C) Images showing the position of such wheeled device at different times. The pneumatic frequency is 2.35 Hz, and the pressure is 128 kPa. (D) Schematic illustration of a wheeled device with a bimetal inner ring and an outer ring made of stainless steel. A PTC heater with a stimulus of 138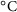 is used to drive the local bending in blister. Demonstration of the heater‐driven wheeled device navigating over (E) stair risers and (F) inclined planes at different times.

The mechanism of blister travelling is further extended to the case of two nested elastic loops. The inner and outer loops are made of PVC sheet and printing paper, respectively, and the values of ratios α and β are 1.05 and 698, respectively. The mismatched length results in the tension and compression in the outer and inner loops, respectively, which drives the simultaneous deformation of both loops and the generation of a well‐defined inner blister (Figure 4A). FEM simulations verify the symmetrical configuration of the blister and the stress distributions in both loops. Furthermore, various configurations of two nested loops placed on a flat table are calculated as a function of α and β using the developed model of Equation (1) considering the loops’ gravity (Figure S7). The analytical configuration illustrates by the dashed line matched well with the experimental result (Figure 4A). It provides helpful guidance of geometrics for studying actuation dynamics in various nested loops.

To drive the blister travelling, eight well‐designed pneumatic actuators ($A_{1}-A_{8}$) for providing local torque are periodically placed in the inner face of inner plastic loop (Figure 4B). A boundary size of 20.37×6.7 cm is illustrated in the nested loops vertically standing on a flat table, and the blister height was 4.03 cm, which echoes well with the analytical results (Figure S8) under the consideration of the actuators’ gravity. Each time an actuator $A_{1}$ reaches the blister's ZCP, it receives a pulse of pneumatic pressure and provides local torque in the inner loop. The local torque induces the occurrence of blister snapping and disturbs the horizontal symmetry of blister. Subsequently, the eccentricity of center‐of‐gravity drives the rolling of whole loops, which is suspended when the pneumatic actuator approaches the bottom position of blister. Meanwhile, the adjacent actuator $A_{2}$ arrives at the ZCP of new blister configuration. We restarts the rolling through pneumatically actuating the actuator $A_{2}$ by another pulse pressure. Therefore, a staggered rolling of the nested loops is generated via a repeated sequence ($A_{1}-A_{2}-\text{…}-A_{7}-A_{8}$) of pneumatic pressure at adjacent actuators. Increasing the frequency of sequential actuation results in the continuous rolling. With a pneumatic frequency at 2.35 Hz and a pressure of 128 kPa, the nested loops rolls as a soft wheel with a translational speed of $v_{w}=14.82\;\mathrm{cm}\;s^{-1}$ (Movie S6, Figure 4C). We could achieve the reverse rolling through adjusting the actuation sequence to the sequence ($A_{7}-A_{6}-\text{…}-A_{1}-A_{8}$). Additionally, the calculated speed distributions of nested loops (Figure S9) matches well with the FEM simulation results under the assumption of no relative slipping between inner and outer loops.

Furthermore, we fabricate a bimetal‐based nested loops as the locomotive wheel. The outer loop is made of stainless steel, and the bimetal inner loop is driven by a PTC heater in the generated blister (Figure 4D). The blister is placed closer to the table, allowing a lower center‐of‐gravity and stable rolling. A stable rolling is observed at a stimulus of $138^{\circ }C$ (Movie S7). It is demonstrated that the wheel is capable of navigating over difficult terrains under the same temperature stimulus. In the first case of stair risers having a unit rise of 1 cm and a unit run of 18.2 cm, the wheel takes 52 s to travel two risers (Figure 4E, Movie S8). Additionally, the wheel exhibits an average velocity of 12.5 $\mathrm{cm}\;\min^{-1}$ on the inclined plane with an uphill angle of 14° (Figure 4F, Movie S9). It suggests the potential of eliminating the complex structure for the wheel maneuvering over obstacles.

## 3 DISCUSSION AND CONCLUSION

To conclude, we have proposed a gearless model with a simple structure of two nested rings to achieve self‐contained rotational actuations that relied on a dynamic buckling mechanism. The continuous snap‐through of buckled blister is driven by a localized change of curvature, and a smaller bending torque is needed to overcome the energy barrier of snaping compared to the widely used bistable beams with discrete states. When the outer ring is rigid, the travelling of blister generated periodic rotational motions, and the rotational speed could be linearly modulated by the amplitude of heating temperature in the bimetallic blister. Even at its fastest, however, the rotational speed only approaches 0.6 rpm, which is limited by thermal conduction rate in the blister. The bimetallic blister‐based device with slow and stable rotation is promising for peristaltically transporting liquids (∼50 $\mu L\;\min^{-1}$). Conventional peristaltic pumps and control valves typically used the electric motor with reduction gearing to match a required rotational speed. However, gearing components not only added the cost but resulted in noise pollution. Soft actuators and robotics for realizing periodic slow rotation have also relied on complex geometries and complicated control systems.

A pneumatically driven elastomeric structure rotated at 15 rpm,^[^ ^15^ ^]^ and commercially electric motors easily achieved speed on the order of 100 to 1000 rpm. The rotational speed in the present design can be enhanced through increasing the rate of inducing the localized change of curvature. Shape memory alloys,^[^ ^19^ ^]^ electroactive polymers,^[^ ^20^ ^]^ electro/magneto‐restrictive materials,^[^ ^21^ ^]^ and macro‐fiber composites (MFC) based on piezoelectric effect^[^ ^22^ ^]^ may be faster strategies to induce curvature change in the inner ring. Through advanced techniques of microfabrication, the miniaturization of buckled‐blisters is also desirable for micro‐switches in micro‐electro‐mechanical systems.^[^ ^23^ ^]^ Due to lower level of energy consumption for the snap‐through, such buckled blister provides an energy‐saving mechanism compared to the switch based on bistable beams.

We develop an analytical model to calculate the stalling torque of blister‐based motor, which is validated against experimental results. The stalling torque is linearly related to the applied bending moment in the blister, and this motor serves as a torque amplifier of more than 15 times. The output torque also depends on the choice of materials, exhibited by the increased stalling torque accompanying an increase in stiffness of the inner loop. The bimetal‐based motor exhibits the capability of outputting a large torque up to 100 mN·m, much larger than that in previous soft rotary actuators (< 50 mN·m).^[^ ^15^ ^]^ At a thermal stimulus of $138^{\circ }C$, the load is lifted up at a constant speed of 0.56 $\mathrm{cm}\;\min^{-1}$, and thus a constant power output of 0.055 mW is calculated. Despite of the rotational invariance of configuration and energy for the buckled blister, the low output power mainly results from large heat dissipation and inelastic deformation in the bimetal strip.

Two nested elastic loops are studied to achieve the rolling‐based locomotive actuation. The locomotor with a customized pneumatic net as local benders achieves a maximum speed of 1 body length per second, faster than the soft robot based on ring oscillator (0.037 body length per second).^[^ ^14^ ^]^ Further, the locomotor driven by heating exhibits the capability of maneuvering over difficult obstacles. Future uses of this work might incorporate an onboard stimulus system as a local bender, for example, MFCs with a built‐in power source and micro‐pneumatic manifold with a compact air compressor.^[^ ^24^ ^]^ Understanding cyclic fatigue and large deformation in the inner loop would be critical challenges for practical applications. It is anticipated that this dynamic mechanism of blister buckling will open new avenues for autonomous robots, energy harvesting and gear‐less motors.

## 4 EXPERIMENTS AND METHODS

### 4.1 Rotator with rigid outer ring

As shown in Figure 2A, a bimetal strip (5J20110, Shanghai Xinxi Alloy Material Co.) with flexivity of 39.48×10^−6^ 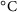 ^−1^, elastic modulus of 113 GPa, length of 50.79 cm, thickness of 0.2 mm and width of 1.5 cm was placed in a plastic outer ring made by 3D‐printing. To facilitate rotation of device, the rotary electrode structure made of copper was used to electrically connect with PTC heater (12 V, 140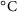, 4–10 W, Weihua Electronics Technology Co.). Room temperature was 25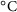. The heating temperature of PTC heater could be tuned by varying the voltage. The fixing position of PTC heater could be changed to adjust the loading positions between *P*_1_ and *P*_5_. When the buckled blister rotated along the outer ring, the screw bearing could transfer the rolling motion to the top plate, which was able to be output for other applications.

As shown in Figure 3E and Figure S6, the rotation behavior of peristaltic device was similar to that of rotary motor, while the rotary electrode structure was modified to leave space for the pump structure. When the top plate rotated, two squeezing rollers fixed on the top plate would press the rubber pipe placed on the surface of the rigid circular wall of rotary electrode. The squeezing mechanism was similar to traditional peristaltic pump, and the fluids in the pipe were periodically driven to flow.

### 4.2 Rolling‐based locomotor

In Figure 4B, two elastic loops were made of polyvinyl chloride (PVC) and paper respectively. The soft pneumatic actuators made of silicone rubber (Dragon Skin 20, Smooth‐On Inc.) were designed based on the Pneumatic Networks structure with some modifications, and the inner loop acted as an inextensible layer of these soft pneumatic actuators. An air pressure control system was assembled to actuate the soft pneumatic actuators by regulating the air pressure from the air compressor. The main components of the control system included solenoid valves (VT307, SMC), electro‐pneumatic regulator (ITV0030, SMC), PLC CPU module (CJ2M‐CPU11, OMRON), PLC digital output module (CJ1W‐OD211, OMRON), and PLC analog output module (CJ1W‐DA08V, OMRON).

In Figure 4D, two elastic loops were made of stainless iron and bimetal (5J20110, Shanghai Xinxi Alloy Material Co.) respectively. To place PTC heater (12V, 140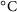, 4‐10 W, Weihua Electronics Technology Co.) at the ZCP of blister, a plate with four wheels and one distancing wheel could keep horizontal when the loops were rolling.

### 4.3 Simulation methods

The FEM simulations were conducted by using commercial software ABAQUS through the explicit procedure. Since the width of blister had a negligible effect on the rotation, the 2D model with linear elastic response was established. The model was discretized by CPE4R elements with reduced integration. To ensure mesh density, the mesh convergence study has been conducted. The kinematic contact between two rings was used in our FEM model since friction had negligible effect. But in the simulation of two nested elastic loops, the penalty contact with the friction coefficient of 0.5 was used. The inner ring was initially in contact with the outer ring and thermal expansion was used to simulate the elongation, so that the preset ratio of mismatched length α was achieved and a buckled blister formed. In simulations, the elastic modulus, Poisson ratios, coefficient of thermal expansion and density of the elastic ring are *E* = 200 GPa, *υ *= 0.3, *γ *= 0.01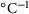 and *ρ *= 7860 $\mathrm{kg}\;m^{-1}$, respectively, unless otherwise denoted. In the FEM model with rigid outer ring, the ratio of the thickness of inner ring to the inner radius of outer ring was 0.042. In the FEM model of two elastic rings, the length and thickness of outer elastic ring are $L_{2}=7.53$ and 0.05 m, respectively, while thickness of inner elastic ring is 0.05 m.

To apply local external torque, the loading region ($\Delta s/L_{2}=4.1\%$) in inner ring was divided into two layers and different temperatures were applied to each layer for inducing local curvature. In Figure 1C, a temperature difference of 0.15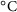 was used to trigger the snapping and the loading period was 0.03 s; In Figure 4A, a temperature difference of 4.5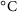 was used to obtain a static buckled blister. In Figures S1–S5, a temperature difference of 1.5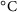 was applied to obtain a static buckled blister; In Figures S1, S3, and S5, a temperature difference of 0.3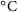 further was used to induce a local torque of 1.87×10^5^ Nm.

## AUTHOR CONTRIBUTIONS

Conceptualization: Baoxiang Shan, Xiangbiao Liao; Methodology: Xiangbiao Liao, Pengfei Yang. Experiment: Pengfei Yang, Xiangbiao Liao, Ruixing Huang; Simulation: Pengfei Yang, Ruixing Huang. Analytics: Fei Dang, Dewen Wang. Visualization: Fei Dang, Hong Liu, Pengfei Yang, Xiangbiao Liao. Supervision: Baoxiang Shan, Xiangbiao Liao. Writing: Xiangbiao Liao, Pengfei Yang, Fei Dang.

## CONFLICT OF INTEREST STATEMENT

The authors declare no conflicts of interest.

## Supporting information

supporting information

supporting information

supporting information

supporting information

supporting information

supporting information

supporting information

supporting information

supporting information

supporting information

## Data Availability

All data are available in the main text or the supplementary materials.
