# Supplementary material for: Motorizing the buckled blister for rotary actuation
Source: Exploration (Beijing). 2024 Mar 14;4(5):20230055. doi: 10.1002/EXP.20230055 (PMC11491305; doi:10.1002/EXP.20230055)
Supplement: Supplementary file 1 — supporting information [file EXP2-4-20230055-s009.docx]

Supporting Information for

Motorizing the buckled blister for rotary actuation

Pengfei Yang^1†^, Ruixing Huang^1†^, Fei Dang^3†^, Baoxiang Shan^5*^, Dewen Wang^1^, Hong Liu^1^, Yi Li^6^, Xiangbiao Liao^2,4*^

**Affiliations**

^1^School of Mechanical Engineering and Automation, Fuzhou University, Fuzhou, 350108, China.

^2^State Key Laboratory of Explosion Science and Technology & Advanced Research Institute of Multidisciplinary Science, Beijing Institute of Technology, Beijing 100081, China.

^3^College of Physics and Electronic Information Engineering, Minjiang University, Fuzhou, 350108, China.

^4^Yangtze River Delta Graduate School of Beijing Institute of Technology, Jiaxing China.

^5^Swave Biotechnology (Suzhou) Co. Ltd, 399 Linquan Road, Room 204, Suzhou 215120, China.

^6^Department of Materials Science and Engineering, University of Connecticut, Storrs, CT, 06268 USA.

^†^These authors contributed equally to this work.

^*^Corresponding email: [bxshan@swave.bio](mailto:bxshan@swave.bio), [liaoxiangbiao@bit.edu.cn](mailto:liaoxiangbiao@bit.edu.cn).

**This PDF file includes:**

Supplementary Materials and Methods

Figure S1 to Figure S11

Legends for Movies S1 to S9

**Other Supplementary Materials for this manuscript include the following:**

Movies S1 to S9

Supplementary Materials and Methods

Analytical models

1. Velocity distributions of the blister confined inside a rigid ring

The large bending stiffness of bimetal sheet leads to little change in blister configuration during the blister travelling. Hence, we assume that the blister maintained the static configuration during blister travelling. The travelling velocity ***v****_a_* was a combined motion of a pure rotation velocity ***v****_r_* around the center of outer ring and a linear motion ***v****_m_* along the blister beam, as shown in Figure 2f. Take the blister in the first quadrant as an example, which travelled in the clockwise direction with the angular velocity $\omega$, for

$\boldsymbol{v}_{r}= \omega l\sin\delta\boldsymbol{e}_{x}-\omega l\cos\delta\boldsymbol{e}_{y},$

$\boldsymbol{v}_{m}=- \omega r\cos\theta\boldsymbol{e}_{x}-\omega r\sin\theta\boldsymbol{e}_{y},$

$\boldsymbol{v}_{a}=\boldsymbol{v}_{m}+\boldsymbol{v}_{r}=(\omega lsin\delta- \omega r\cos\theta)\boldsymbol{e}_{x}-(\omega r\sin\theta+\omega l\cos\delta)\boldsymbol{e}_{y}$, (S1)

where $\theta$ represents deflection angle, $l=\sqrt{x^{2}+y^{2}}$is the distance related to the origin of coordinates, $\delta=\arctan\left( \frac{y}{x} \right)$ and ***e****_x_* and ***e****_y_* are unit vectors in x and y axes respectively. The result is verified by FEM simulations.

The angular velocity $\omega$ of blister-based rotator has the following relationship with respect to the velocity at the loading point *v*_load_:

$\omega=\frac{v_{load}}{\sqrt{\left( r\cos\theta-l\sin\delta\right)^{2}+\left( r\sin\theta+l\cos\delta\right)^{2}}}$ (S2)

Angular velocity $\omega$ can be normalized as

$\bar{\omega}=\frac{\omega}{\frac{v_{load}}{r}}$. (S3)

1. Calculations of stalling force

In the rotary motor, the screw bearing transfers the rotational motion to the top plate. When the stalling force $F_{stall}$ is applied at the edge of top plate, the rotator device keeps stationary and a force $F_{bearing}$ is transited to the blister, as shown in **Figure S10**. The top plate satisfies the balance equation:

$F_{stall}r_{stall}=F_{bearing}r_{bearing}$, (S4)

while the blister was equilibrated with *F*_bearing_ and local torque *M*.

The blister can be divided into two segments by the loading position. For the left segment, the equilibrium equation for the red region with local torque is

$K\left( \theta^{'}(s)+\frac{M}{K} \right)^{'}-F_{x1}\sin\theta(s)+F_{y1}\cos\theta(s)=0$, (S5)

while the equilibrium equation in other segment without local torque is

$K\theta^{''}(s)-F_{x1}\sin\theta(s)+F_{y1}\cos\theta(s)=0$. (S6)

For the right segment, the equilibrium equation can be expressed as

$K\theta^{''}(s)-F_{x2}\sin\theta(s)+F_{y2}\cos\theta(s)=0$. (S7)

In the whole blister curve, the deflection angle and curvature are continuous. Since the loading region contacts with the stationary PTC heater, $F_{bearing}$ can be obtained

$F_{bearing}=\sqrt{\left( F_{x2}-F_{x1} \right)^{2}+\left( F_{y2}-F_{y1} \right)^{2}}$. (S8)

1. Analytical configuration in two nested loops

Napoli *et al*.^23^ derived the balance equation of two nested elastic rings. With the consideration of gravity, the equilibrium equations can be given by

$$K_{\text{in}}\theta_{\text{in}}^{''}(s_{\text{in}})-F_{x}^{\text{in}}\sin\theta_{\text{in}}(s_{\text{in}})+F_{y}^{\text{in}}\cos\theta_{\text{in}}(s_{\text{in}})=0,$$

$$K_{\text{out}}\theta_{\text{out}}^{''}(s_{\text{out}})-F_{x}^{\text{out}}\sin\theta_{\text{out}}(s_{\text{out}})+F_{y}^{\text{out}}\cos\theta_{\text{out}}(s_{\text{out}})=0,$$

$K_{\text{com}}\theta_{\text{com}}^{''}(s_{\text{com}})-F_{x}^{\text{com}}\sin\theta_{\text{com}}(s_{\text{com}})+F_{y}^{\text{com}}\cos\theta_{\text{com}}(s_{\text{com}})=0,$ (S9)

where subscript/superscript “in”, “out” and “com” refer to the parts of inner ring, outer ring and their adhered region, respectively; {*K*_in_=*E*_in_*I*_in_, *K*_out_=*E*_in_*I*_in_, *K*_com_=*E*_com_*I*_com_}are the bending rigidity; {*E*_in_, *E*_out_, *E*_com_} represent the elastic modulus; {*I*_in_, *I*_out_, *I*_com_} are the moment of inertia in the beam cross-section. $\{\theta_{\text{in}}\left( s_{\text{in}} \right), \theta_{\text{out}}\left( s_{\text{out}} \right),\theta_{\text{com}}(s_{\text{com}})$} represents the deflection angles relative to the x axis, $\{s_{\text{in}}, s_{\text{out}},s_{\text{com}}$} represent the arc length; $\{F_{x}^{\text{in}}$, $F_{x}^{\text{out}}$, $F_{x}^{\text{com}}\}$ denote the internal forces in the x direction, and {$F_{y}^{\text{in}}=\rho_{\text{in}}gs_{\text{in}}$,$F_{y}^{\text{out}}=\rho_{\text{out}}gs_{\text{out}}$, $F_{y}^{\text{com}}=\left[ \rho_{\text{in}}\bar{s}_{\text{in}}+\rho_{\text{out}}\bar{s}_{\text{out}}+\rho_{\text{com}}\left( \bar{s}_{\text{com}}-s_{\text{com}} \right) \right]g$} denote the internal forces in the y direction induced by gravity and $\rho$ represents the density (Figure S11). For the two nested elastic rings, deflection angles and curvatures are continuous along the beam. At the detachment points,

$s_{\text{com}}=\bar{s}_{\text{com}}$, $\theta_{\text{com}}^{'}\left( \bar{s}_{\text{com}} \right)=\theta_{\text{in}}^{'}\left( \bar{s}_{\text{in}} \right)=\theta_{\text{out}}^{'}\left( \bar{s}_{\text{out}} \right)$,

$\theta_{\text{com}}\left( \bar{s}_{\text{com}} \right)=\theta_{\text{in}}\left( \bar{s}_{\text{in}} \right)=\theta_{\text{out}}\left( \bar{s}_{\text{out}} \right)$ (S10)

At the ground detachment point

$s_{\text{com}}=0$, $\theta_{\text{com}}^{'}\left( 0 \right)=0$ and $\theta_{\text{com}}\left( 0 \right)=0$ . (S11)

By solving equations (S9) numerically, the configuration of two nested rings with different parameters can be obtained (**Figure S7**).

1. Velocity distributions for locomotive wheel

The rolling of two nested elastic rings is a combined motion of a translational motion along the horizontal table and a motion along the beam. The result is shown in Figure S9, verified by FEM simulations. The translational velocity of wheeled device is

$v=\frac{v_{load}}{2\cos\frac{\theta}{2}}$, (S12)

which can be normalized as $\bar{v}=\frac{v}{v_{load}}$.

**Supplementary Figures**


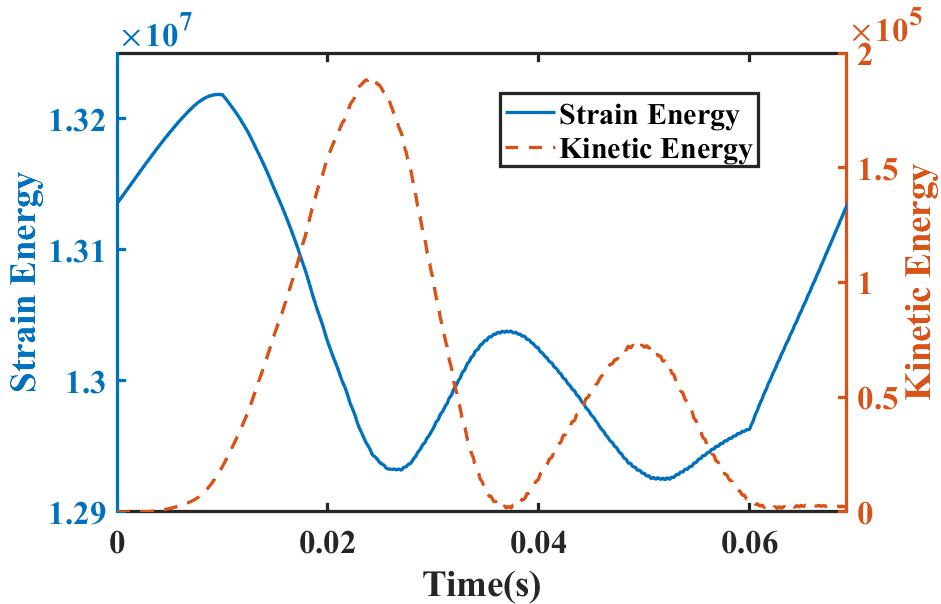


Figure S1. The changes of strain and kinetic energies as a function of time during one cycle of loading-unloading the local bending moment, calculated by FEM simulations. The unit of energies is Joule.


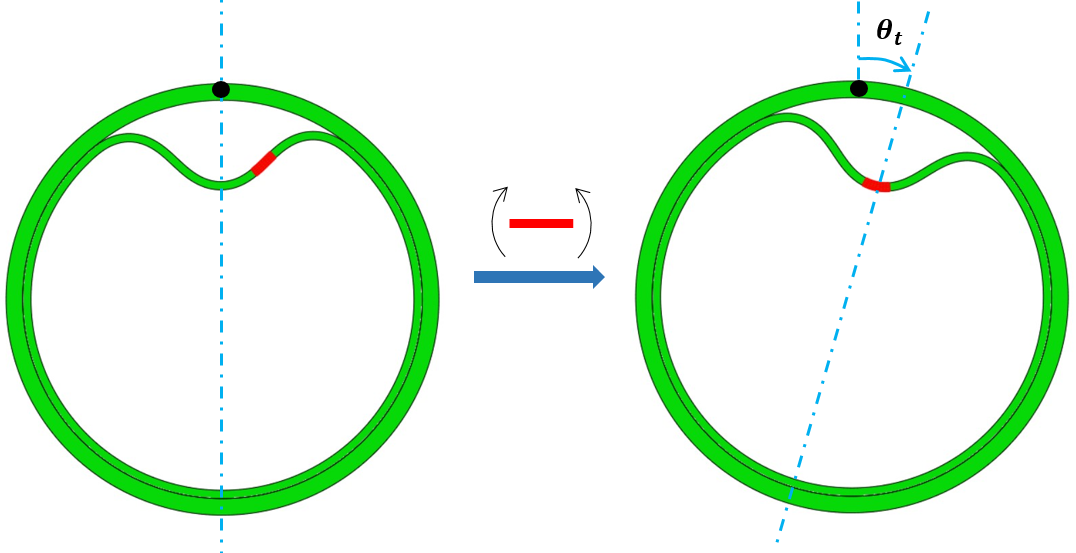


Figure S2. The transition of blister configuration and occurrence of blister snapping with applying a local bending at the local region (red).


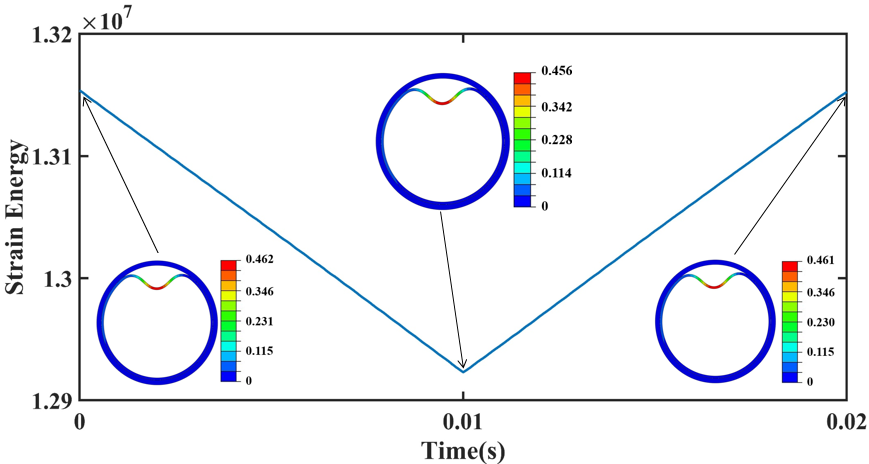


Figure S3. The change of strain energy (unit: Joule) in the inner ring with applying and unloading a local bending at the position $\boldsymbol{P}_{\boldsymbol{5}}$, that is the apex of blister. Insets showed distributions of vertical displacement (unit: m) in the blister. With loading the bending moment, the blister only moved up with decreasing strain energy, but no rotation was observed.


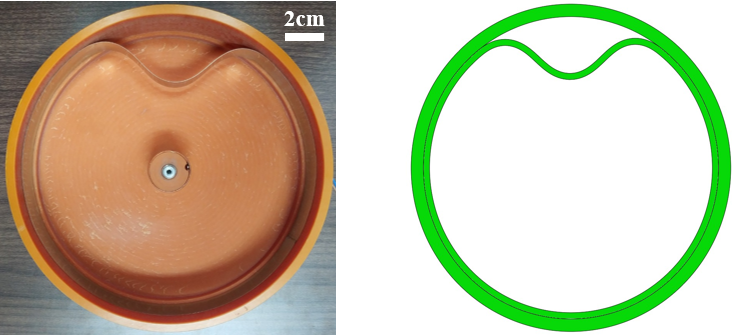


**Figure S4.** The qualitive comparison of static configurations between experimental and simulated blisters.


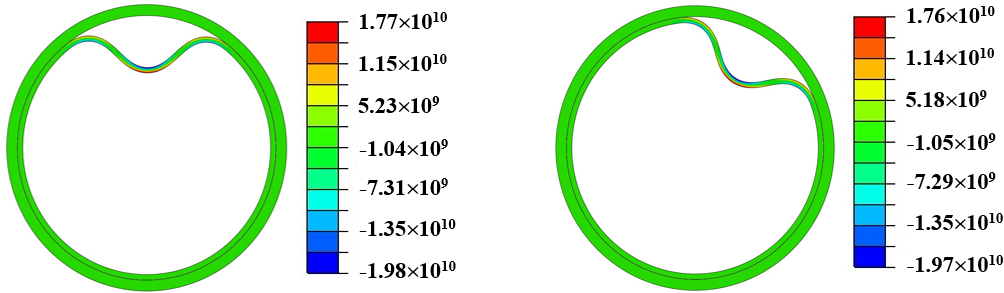


**Figure S5.** Distributions of stress along the beam direction for the blister before and after the occurrence of snapping. In this snapping transition, the local bending was maintained.


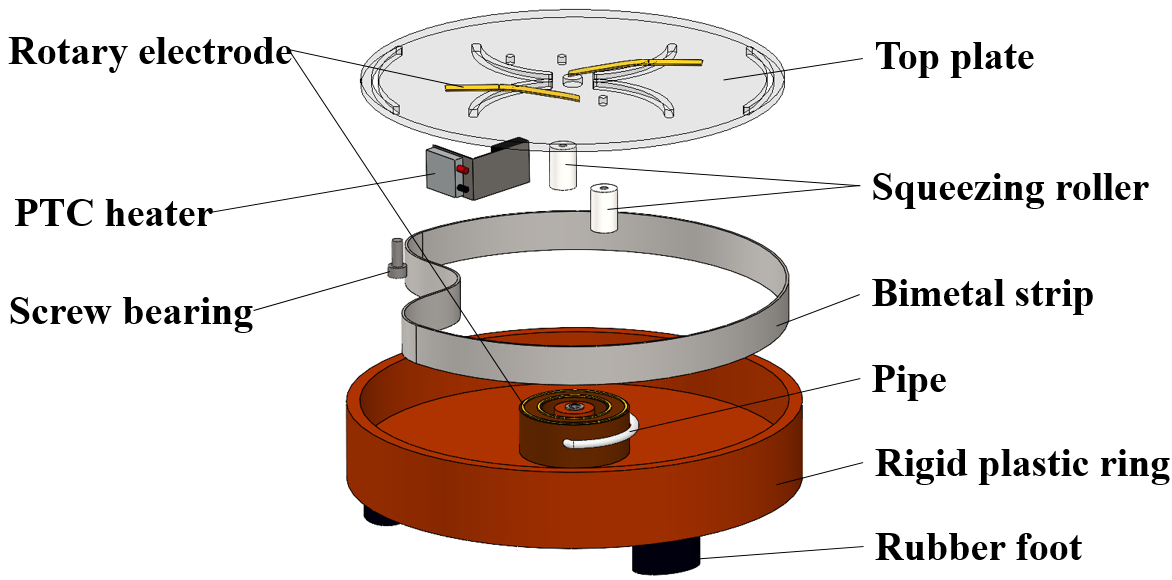


**Figure S6.** Layer-by-layer illustration of a peristaltic pump using the rotation of buckled blister. A PCT heater was used as the source of local stimulus, and a screw bearing was capable of transferring rotation to the top plate. Once the blister rotating, the roller fixed on the top plate could cyclically squeeze the rubber pipe filled with water, resulting in water periodically flowing.


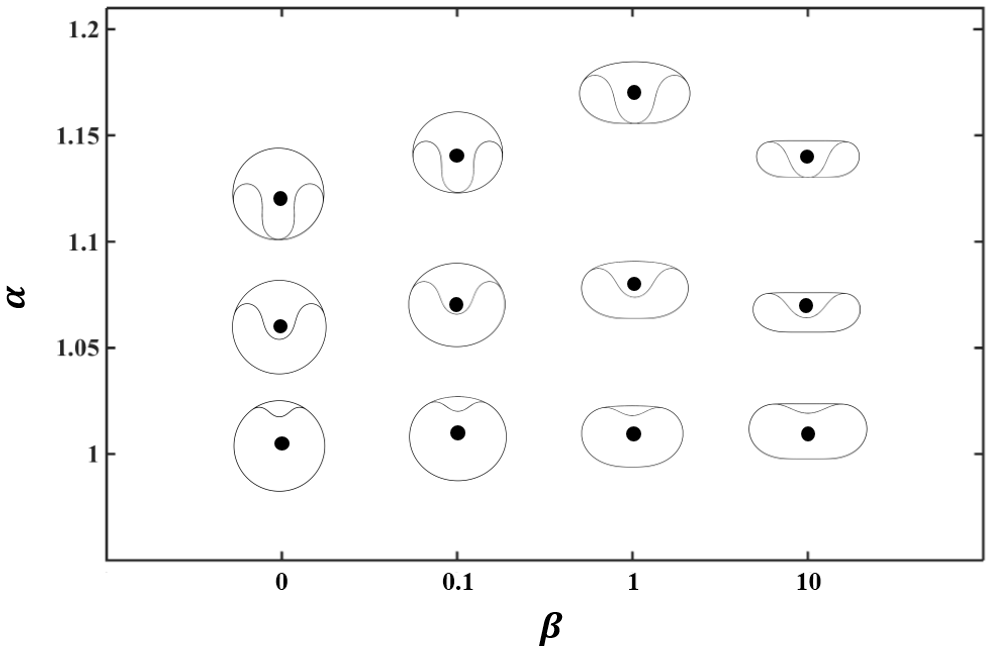


**Figure S7.** Analytically calculated diagram of equilibrium configurations of two nested elastic loops for several values of $\alpha$ and $\beta$. The gravity of loops was 0.754 N in the calculations.


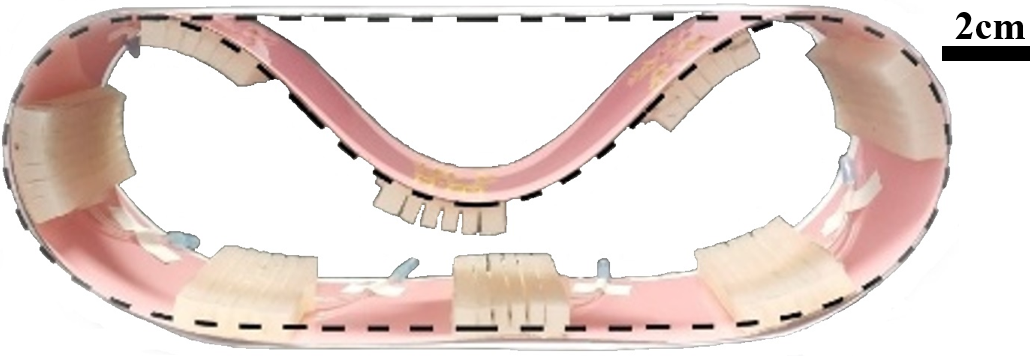


**Figure S8.** Geometric comparison of blisters with pneumatic actuators between the experimental configuration and analytically calculated configuration (dash line) at equilibrium. The gravity of loops and all pneumatic actuators of 0.754 N was considered in the calculations. It illustrated the analytical model could well predict the geometric configurations.


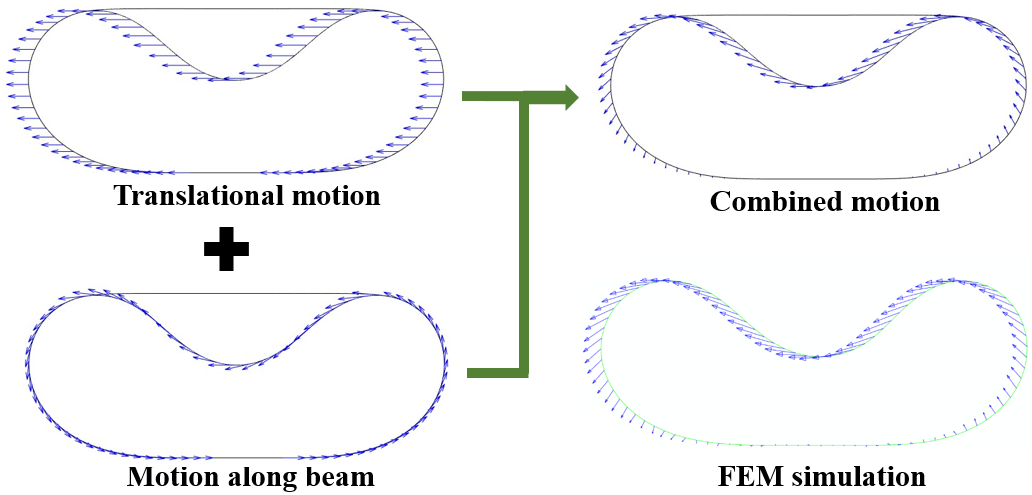


**Figure S9.** Speed distributions of analytically and numerically calculated values in the locomotive device with two nested loops. The speed in the inner loop could be divided into two components of the purely translational motion along the table and the motion along the inner loop. The numerically result from FEM simulation matched well with the analytical result.


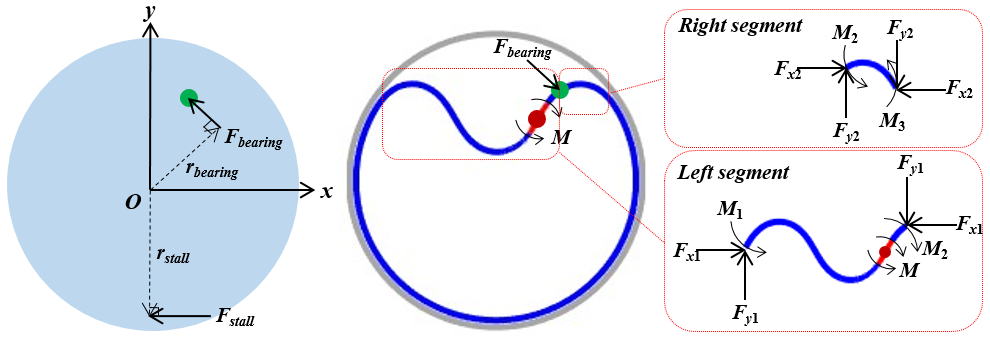


**Figure S10.** Schematics of balanced forces in rotary motor device. The top plate was equilibrated with $F_{stall}$ and $F_{bearing}$, while the blister was equilibrated with $F_{bearing}$ and local torque *M*.


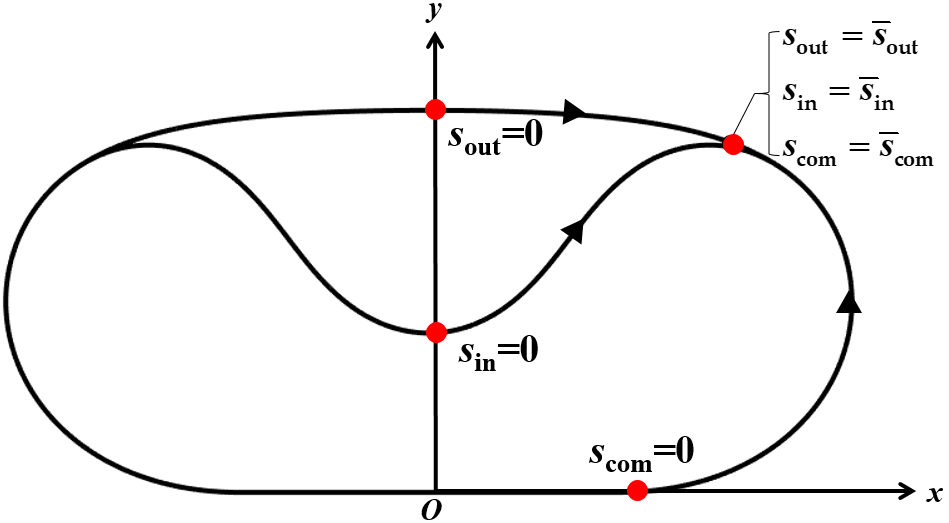


**Figure S11.** Schematic of equilibrium configuration in two nested elastic rings with consideration of rings’ gravity.

**Supplementary Movies**

Movie S1. Travelling of buckled blister driven by continuous heating stimulus in FEM simulation.

Movie S2. Experimental observation of blister rotating confined in a rigid ring.

Movie S3. Pause and resuming of the blister travelling via controlling the heating stimulus in FEM simulation.

Movie S4. A rotary motor lifting a weight of 59 g based on the mechanism of blister rotating.

Movie S5. The blister-based rotary motor serving as a peristaltic pump for pumping water.

Movie S6. The locomotive motion in wheeled device made of PVC /paper loops and driven by pneumatic actuators.

Movie S7. The locomotive motion in wheeled device made of bimetal/stainless steel loops and driven by heating stimulus.

Movie S8. The wheeled device with a bimetal blister navigating across an obstacle of stair risers.

Movie S9. The wheeled device with a bimetal blister navigating across an obstacle of inclined plane.
